# STABILIZING ROLE OF HCN CHANNELS ON POST-CAMP MECHANISMS OF DETRUSOR MYOCYTE CONTROL

**DOI:** 10.1093/geroni/igac059.2448

**Published:** 2022-12-20

**Authors:** Ramalakshmi Ramasamy, Alya AlObaidi, Phillip Smith

**Affiliations:** UConn Health, Farmington, Connecticut, United States; U Hart, Hartford, Connecticut, United States; UConn Health, Farmington, Connecticut, United States

## Abstract

Aging is associated with an increased incidence of co-morbidities, including detrusor underactivity (DU). DU is defined as the failure to create sufficient and durable expulsive force to adequately empty the urinary bladder during a normal voiding timespan. DU is prevalent in older adults, as evidenced by its prevalence in nearly two-thirds of nursing home residents. Current treatments are mostly palliative or come with many side effects. β-adrenoceptor-mediated relaxation is the primary mechanism of detrusor relaxation, and Hyperpolarization-activated Cyclic Nucleotide-gated (HCN) channels have previously been identified by us and others as a very important mediator of this relaxation, however the role of HCN in detrusor relaxation has not been elucidated. Hence, we seek to characterize its role in adrenergic relaxation mechanisms and spontaneous myocyte activity. Male and female 10–12-month-old C57Bl/6 mice were used for this study. Pharmacomyography studies were performed to assess the effect of different drugs that act at various steps along the adrenergic relaxation pathway, +/- CsCl, an HCN blockade at [5mM]. As expected, we saw that increasing HCN opening probability by isoproterenol or forskolin (adenylyl cyclase/cAMP-agonist) or lamotrigine (HCN-activator) resulted in decreases in tonic tension, but were diminished in the presence of CsCl. Mechanisms modulated by H89 (PKA-inhibitor) and NS1619 (BK-channel-agonist) show no change in tonic tension, however spontaneous phasic activity significantly increases. These data support increased cAMP, not hyperpolarization, as the key inductor of HCN in adrenergic relaxation.

